# Spatiotemporal Change of Net Primary Productivity and Its Response to Climate Change in Temperate Grasslands of China

**DOI:** 10.3389/fpls.2022.899800

**Published:** 2022-05-24

**Authors:** Rong Ma, Chunlin Xia, Yiwen Liu, Yanji Wang, Jiaqi Zhang, Xiangjin Shen, Xianguo Lu, Ming Jiang

**Affiliations:** ^1^Northeast Institute of Geography and Agroecology, Chinese Academy of Sciences, Changchun, China; ^2^College of Mapping and Geographical Sciences, Liaoning Technical University, Fuxin, China; ^3^University of Chinese Academy of Sciences, Beijing, China

**Keywords:** temperate grassland, vegetation, NPP, climate change, China

## Abstract

The temperate grasslands in China play a vital part in regulating regional carbon cycle and climate change. Net primary productivity (NPP) is a crucial index that reflects ecological function of plants and the carbon sequestration capacity of grassland ecosystem. Climate change can affect NPP by changing vegetation growth, but the effects of climate change on the NPP of China’s temperate grasslands remain unclear. Based on MODIS data and monthly climate data during 2000–2020, this study explored the spatiotemporal changes in grassland NPP and its response to climate change in temperate grasslands of China. We found that the annual NPP over the entire China’s temperate grasslands increased significantly by 4.0 gC/m^2^/year from 2000 to 2020. The annual NPP showed increasing trends for all the different grassland vegetation types, with the smallest increase for temperate desert steppe (2.2 gC/m^2^/year) and the largest increase for temperate meadow (5.4 gC/m^2^/year). The correlation results showed that increased annual precipitation had a positive relationship with the NPP of temperate grasslands. Increased summer and autumn precipitation could increase grassland NPP, particularly for the temperate meadow. With regard to the effects of temperatures, increased temperature, particularly the summer maximum temperature, could decrease annual NPP. However, increased spring minimum temperature could increase the NPP of temperate desert steppe. In addition, this study found, for the first time, an asymmetric relationship between summer nighttime and daytime warming and the NPP of temperate meadow. Specifically, nighttime warming can increase NPP, while daytime warming can reduce NPP in temperate meadow. Our results highlight the importance of including seasonal climate conditions in assessing the vegetation productivity for different grassland types of temperate grasslands and predicting the influences of future climate change on temperate grassland ecosystems.

## Introduction

Grassland ecosystems play a key role in regulating the carbon cycle and maintaining ecological balance (Bengtsson et al., 2019; Hoover et al., 2022; Shen et al., 2022a,b). Net primary productivity (NPP) is not only a crucial indicator of carbon sequestration (Zhang et al., 2016; Hossain et al., 2021), but also a key reference for assessing grassland ecosystem function (Seeber et al., 2022; Wang et al., 2022). Previous studies have shown that climate change has significant effects on grassland NPP (Liu et al., 2021; Xiong et al., 2021). In the past few decades, global grassland NPP has undergone significant changes owing to climate change (Cao et al., 2020; Li et al., 2022), which may affect the ecological environment and carbon cycle in the grassland regions (Shen et al., 2020). Understanding spatiotemporal changes in grassland NPP and its response to climate change is essential for predicting grassland carbon storage capacity and clarifying the relationship between vegetation and climate.

Temperate grasslands of China are the third largest grassland region and play a crucial role in regional carbon cycle (Piao et al., 2006). As a sensitive region to climate change, the grassland vegetation in this region has changed dramatically due to the impact of climate change (Dangal et al., 2016; Jiang et al., 2020; Zhang et al., 2020, 2021). Many scholars have studied the relationship between climate change and the NPP of China’s temperate grasslands (Yu et al., 2021). For example, Yan et al. (2021) reported that grassland NPP in Horqin is positively related to precipitation. Zhang et al. (2019) and Wu et al. (2021) indicated that precipitation is a determining factor affecting grassland NPP in Xilingol and Hulunbuir. Nanzad et al. (2021) concluded that the grassland NPP of central Inner Mongolia is affected by precipitation and temperature. However, these previous studies mostly concentrated on the influence of climate change on NPP for a specific grassland type at a local scale. As climate change could have a distinct influence on grassland NPP for different vegetation types in different areas (Gang et al., 2014; Xu et al., 2022), it is essential to analyze the effects of climate change on NPP for different types of grassland over the entire temperate grasslands in China. Recently, Guo et al. (2021) found that the NPP of Inner Mongolian grasslands was significantly affected by rainfall in summer and temperature in autumn. Zhang et al. (2019) indicated that spring temperature had a positive relationship with grassland NPP in Hulunbuir, whereas summer temperature had a negative correlation with NPP. As grassland vegetation NPP may have different responses to seasonal climate change (Lei et al., 2020), the effects of seasonal climate changes on NPP of China’s temperate grasslands need to be further studied. An interesting work found that the relationships between daytime and nighttime temperatures and grassland productivity in the Tibetan Plateau were asymmetric (Shen et al., 2015). Specifically, nighttime warming could inhibit the growth of grassland vegetation, while daytime warming could promote grassland vegetation growth in the Tibetan Plateau (Shen et al., 2017). Unlike the wet and cold Tibetan Plateau regions, the climate of China’s temperate grasslands is relatively dry and warm. However, it is unclear whether the nighttime and daytime temperatures have distinct impacts on temperate grassland productivity in China. In the context of global asymmetry in nighttime and daytime warming (Liu et al., 2004; Shen et al., 2014), it is necessary to explore the influence of temperature during day and night on NPP of China’s temperate grasslands.

Based on MODIS NPP and meteorological data from 2000 to 2020, we studied the spatial and temporal changes of the NPP for different types of temperate grassland and their relationships with meteorological factors (including precipitation, maximum temperature, average temperature, and minimum temperature). Clarifying spatial-temporal variations of grassland NPP and its response to climate change can contribute to further understand the grassland vegetation change and advance our understanding of fundamental processes in plant biology of temperate grasslands in northern China. Since the China’s temperate grassland region is an arid and semi-arid region, we put forward the following hypotheses: (1) the NPP of temperate grasslands in this region is significantly positively correlated with precipitation; (2) the increase of daytime temperature in the warm season may reduce the grassland NPP by enhancing water evaporation in the arid regions of the study area.

## Data and Methods

### Study Area

**Figure 1:**
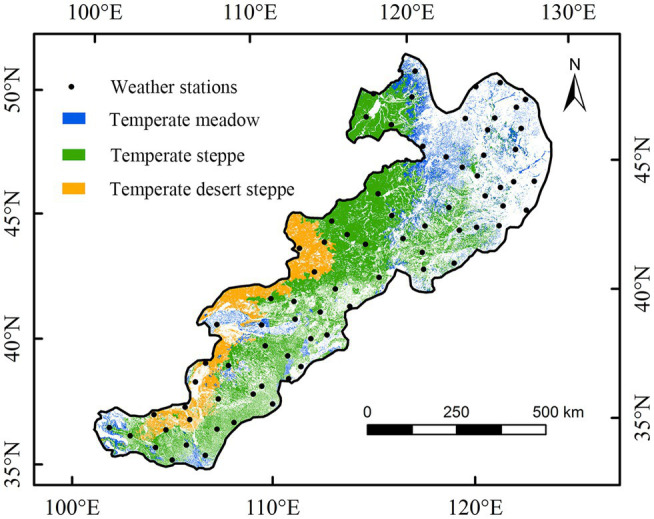
Distributions of grassland vegetation and meteorological stations in China’s temperate grasslands.

China’s temperate grassland is distributed in the Loess Plateau, Inner Mongolia Plateau and Songliao Plain (Shen et al., 2018). The climate of study area is an arid and semi-arid climate, and grassland vegetation in this region includes temperate steppe, desert steppe, and meadow (Figure 1; Shen et al., 2016, 2018). These different vegetation types are classified based on vegetation coverage and physical geographic environment (Piao et al., 2006). The vegetation coverage of temperate desert steppe, steppe, and meadow is about 5–10%, 10–30%, and >30%, respectively (Shen et al., 2015). Owing to the fragile ecological environment of these temperate grasslands, the study area is very sensitive to climate change (Bao et al., 2019; Bai et al., 2021). Temperate desert grassland is mainly concentrated in the west of the study area, with annual mean temperature of 7.2°C and annual precipitation of 45–215 mm (Hossain et al., 2021). Temperate steppe is the most widely distributed and representative of the various grassland types. The annual average temperature is about 3.0°C, and the annual rainfall is 300–400 mm (Zhao et al., 2022). Temperate meadows are mainly concentrated in the northeast part of China’s temperate grassland region, with annual mean temperature of −2.2°C and an annual precipitation of 350–500 mm (Wang et al., 2020). The temperate meadow area in northeast study area has a semi-humid monsoon climate.

### Dataset

In this study, MOD17A3HGF NPP data from 2000 to 2020 were provided by Earth Science Data Systems,^1^ with a spatial resolution of 500 m. These data have undergone a series of rigorous technical processes with high accuracy and have been widely used by a large number of scholars (Ma et al., 2021; Gang et al., 2022). Meteorological data (2000–2020) used in this study were drawn from 76 weather stations (Supplementary Table 1) in China’s temperate grasslands, including monthly precipitation, maximum, mean, and minimum temperatures. These climate data were obtained from National Meteorological Science Data Center.^2^ According to previous studies (Peng et al., 2013; Shen et al., 2018), this study used the maximum and the minimum temperatures to represent the daytime and nighttime temperatures, respectively. The land use data used in this study were obtained from the Resources & Environment Science & Data Centre.^3^ It has a spatial resolution of 100 m × 100 m, including 25 secondary land-use classifications and six primary land-use classifications.

### Method

The meteorological data were spatially interpolated based on the inverse distance weighted method and then re-sampled into monthly meteorological data with a spatial resolution consistent with NPP data (Mueller et al., 2004). To effectively exclude the influence of land type transformation, we extracted unchanged grassland during the study period, using two phases of land use data in the study area. The NPP for each grassland type and the meteorological factor values were the corresponding mean values of all pixels in the study area. Based on previous studies (Piao et al., 2011), this study defines the seasons of the study area as winter (December–February), autumn (September–October), summer (June–August), and spring (March–May). The value of the meteorological elements in each season was the average value of the corresponding months. Simple linear regression was adopted to investigate the changing trend invariables over 21 years (Zhuang et al., 2022):


(1) $$\mathrm{slope}=\frac{\left(n\times \sum_{t=1}^{n}t\times X_{t}\right)-\left(\sum_{t=1}^{n}t\sum_{t=1}^{n}X_{t}\right)}{n\times \sum_{t=1}^{n}t^{2}-{\left(\sum_{t=1}^{n}t\right)}^{2}}$$

where *slope* represents the tendency of NPP or meteorological factors for each pixel. If *slope* > 0 (<0), it indicates that the NPP or meteorological factor of the element has an increasing (a decreasing) trend, *n* represents the number of years (21 years), *t* is the serial number of a year in 21 years, and X*_t_* represents the long-term averaged NPP (meteorological factors).

## Results

### Spatiotemporal Variation of NPP for China’s Temperate Grasslands

**Figure 2:**
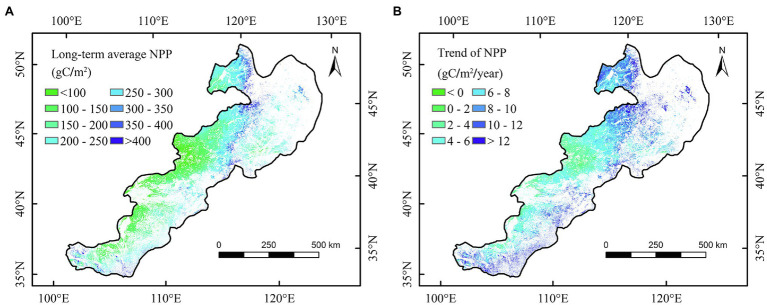
Long-term average **(A)** and temporal trends **(B)** in annual net primary productivity (NPP) of China’s temperate grasslands during 2000–2020.

**Figure 3:**
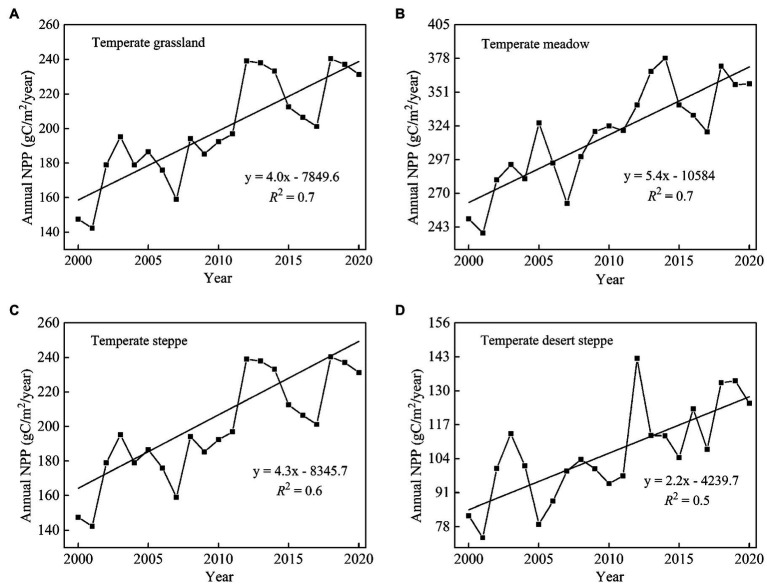
Temporal trends in annual net primary productivity (NPP; gC/m^2^/year) of temperate grassland **(A)**, temperate meadow **(B)**, temperate steppe **(C)**, and temperate desert steppe **(D)** in China’s temperate grassland region. during 2000–2020.

We found that the long-term average annual NPP of China’s temperate grasslands was approximately 198.7 gC/m^2^ during 2000–2020 (Figure 2A). The long-term average annual NPP was approximately 106.1, 206.8, and 316.9 gC/m^2^ for temperate desert steppe, steppe, and meadow, respectively. In terms of temporal change, the average annual NPP over the entire temperate grasslands in China increased significantly (*p* < 0.05) by 4.0 gC/m^2^/year from 2000 to 2020 (Figure 3A). The annual NPP showed the largest increase (5.4 gC/m^2^/year; *p* < 0.05) for temperate meadow and the smallest increase (2.2 gC/m^2^/year) for temperate desert steppe (Figures 3B,C). At the spatial scale, an obvious increase in NPP was concentrated in eastern part of the temperate grassland region (Figure 2B). In contrast, a weak decrease in NPP was observed in west part of the study area (Figure 2B).

### Correlations Between NPP of China’s Temperate Grasslands and Climate Factors

To examine the effects of seasonal climate change on the NPP of temperate grasslands, we analyzed the correlations between the NPP and meteorological factors during the period of 2000–2020 (Table 1). The results showed that the annual NPP had a significant (*p* < 0.05) positive relationship with the annual precipitation (*p* < 0.01). Moreover, the annual NPP had positive correlations with precipitation in both summer and autumn (Table 1). The annual NPP showed a positive correlation with temperatures in spring but a negative correlation with temperatures in the other seasons, with a significant (*p* < 0.05) negative relationship with summer maximum temperature (Table 1).

**Table 1:** Correlation coefficients between climate variables and mean net primary productivity (NPP) over China’s temperate grasslands during 2000–2020.

	Precipitation	Mean temperature	Maximum temperature	Minimum temperature
Annual	0.830^**^	−0.301	−0.146	−0.169
Spring	0.284	0.134	0.373	0.215
Summer	0.720^**^	−0.383	−0.484^*^	−0.085
Autumn	0.496^*^	−0.017	−0.188	−0.125
Winter	−0.213	−0.247	−0.201	−0.270

Significant at ^*^p < 0.05 and ^**^p < 0.01 levels (same below).

We also explored the correlations between grassland NPP and seasonal climate factors during 2000–2020 (Table 2). The NPP of temperate grasslands had a positive relationship with annual precipitation. For both temperate meadow and temperate desert steppe, we found the increase of summer and autumn precipitation was linked to the increase of the annual NPP. By contrast, the annual temperature was negative correlated with grassland NPP for different vegetation types (Table 2). For seasonal climate effects, the grassland NPP of different vegetation types was positively correlated with spring temperature. The positive relationship between the annual NPP and the minimum temperature in spring was significant for temperate desert steppe. In summer, we observed an asymmetric relationship between maximum and minimum temperatures and the NPP of the temperate meadow. The annual NPP of the temperate meadow was positively related to the minimum temperature, but was significantly (*p* < 0.05) and negatively related to the maximum temperature (Table 2).

**Table 2:** Correlation coefficients between climate variables and net primary productivity (NPP) for different grassland types of China’s temperate grasslands during 2000–2020.

	Precipitation	Mean temperature	Maximum temperature	Minimum temperature
*Annual*				
Temperate meadow	0.838^**^	−0.238	−0.235	−0.169
Temperate steppe	0.847^**^	−0.345	−0.192	−0.192
Temperate desert steppe	0.805^**^	−0.251	−0.091	−0.228
*Spring*				
Temperate meadow	0.386	0.147	0.111	0.069
Temperate steppe	0.280	0.072	0.311	0.151
Temperate desert steppe	0.314	0.387	0.438	0.478^*^
*Summer*				
Temperate meadow	0.722^**^	−0.333	−0.484^*^	0.243
Temperate steppe	0.729^**^	−0.440	−0.523^*^	−0.137
Temperate desert steppe	0.738^**^	−0.386	−0.526^*^	−0.188
*Autumn*				
Temperate meadow	0.476^*^	−0.006	−0.009	0.136
Temperate steppe	0.497^*^	−0.137	−0.167	−0.094
Temperate desert steppe	0.349	−0.312	−0.193	−0.345
*Winter*				
Temperate meadow	−0.100	−0.126	−0.221	−0.176
Temperate steppe	−0.166	−0.247	−0.204	−0.260
Temperate desert steppe	−0.124	−0.229	−0.241	−0.321

Significant at ^*^p < 0.05 and ^**^p < 0.01 levels.

**Figure 4:**
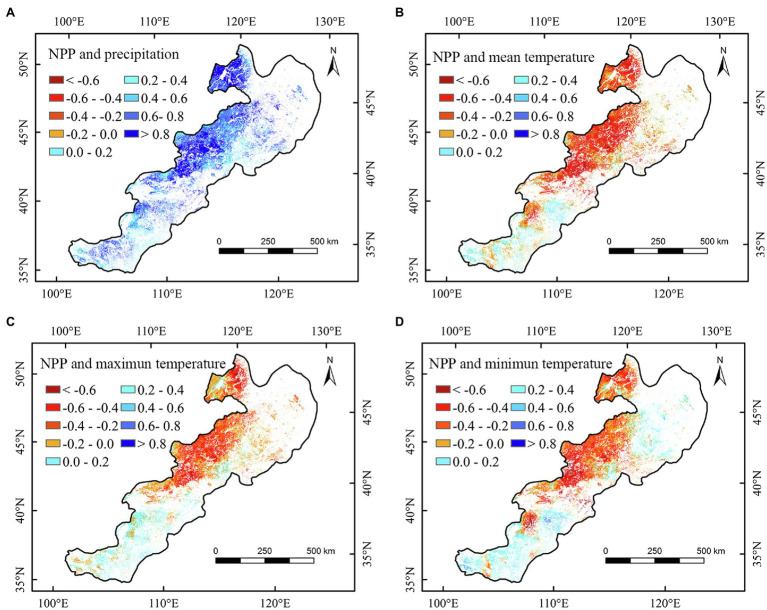
Correlations between annual net primary productivity (NPP) and annual precipitation **(A)**, annual mean temperature **(B)**, annual maximum temperature **(C)**, and annual minimum temperature **(D)** of temperate grasslands in China during 2000–2020.

**Figure 5:**
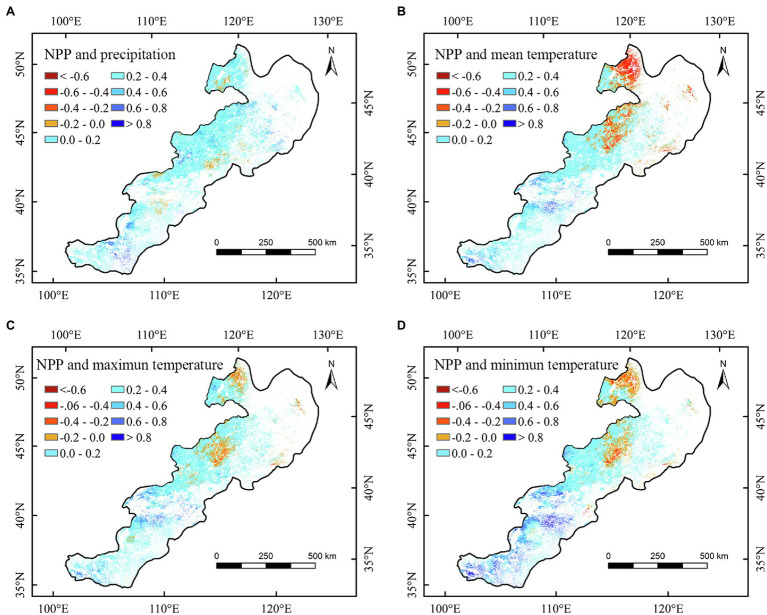
Correlations between annual net primary productivity (NPP) and spring precipitation **(A)**, spring mean temperature **(B)**, spring maximum temperature **(C)**, and spring minimum temperature **(D)** of temperate grasslands in China during 2000–2020.

**Figure 6:**
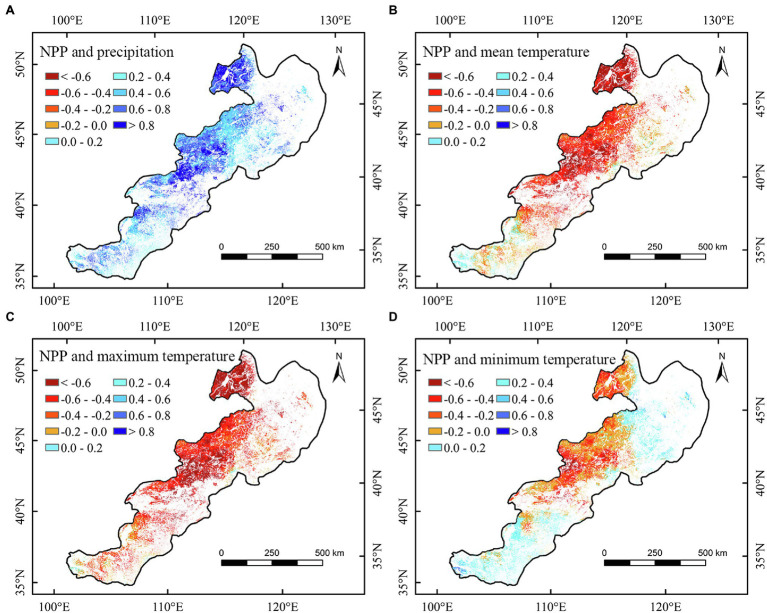
Correlations between annual net primary productivity (NPP) and summer precipitation **(A)**, summer mean temperature **(B)**, summer maximum temperature **(C)**, and summer minimum temperature **(D)** of temperate grasslands in China during 2000–2020.

Spatially, the correlation between the annual NPP of different regions and climate factors showed apparent spatial heterogeneities (Figures 4–6 and Supplementary Figures 1, 2). We found that the annual NPP of temperate meadow had a positive relationship with annual precipitation and a negative relationship with annual temperature in the northeast temperate grassland region (Figures 4A–D). The positive correlation between the NPP of temperate steppe and annual temperature was mainly concentrated in the southwest of the study area (Figures 4A–D). In different seasons, the annual NPP showed a significant (*p* < 0.05) positive relationship with summer and autumn precipitation in the central temperate grassland region (Figure 6A; Supplementary Figure 1A). For the northeastern region, the annual NPP was negatively and positively related to the summer maximum and minimum temperatures, respectively (Figures 6C,D).

**Figure 7:**
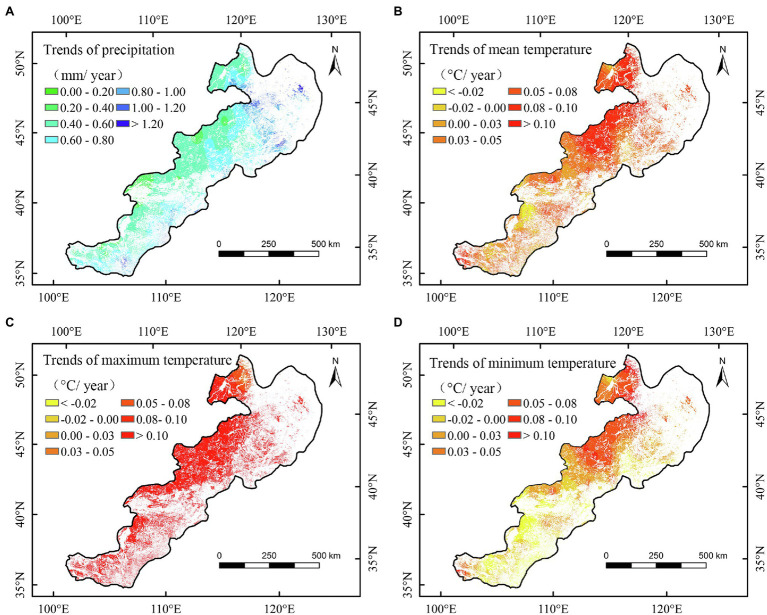
Spatial variation trends in annual precipitation **(A)**, annual mean temperature **(B)**, annual maximum temperature **(C)**, and annual minimum temperature **(D)** of temperate grasslands in China during 2000–2020.

To further explain the changes in NPP of temperate grasslands, we calculated spatiotemporal variations in meteorological factors from 2000 to 2020 (Table 3 and Figure 7). The annual precipitation and temperature showed increasing trends (Table 3). In different seasons, both spring and summer minimum temperatures showed an increasing trend, but minimum temperatures in autumn and winter decreased during the study period (Table 3). The maximum temperature increased in spring, but decreased in the other seasons (Table 3). With regard to variations in seasonal precipitation, spring and summer precipitation showed increasing trends (Table 3).

**Table 3:** Temporal trends in temperatures (°C/year) and precipitation (mm/year) of temperate grasslands in China during 2000–2020.

	Precipitation	Mean temperature	Maximum temperature	Minimum temperature
*Annual*				
Temperate grasslands	0.354^*^	0.001	0.017	0.009
Temperate meadow	0.514^**^	−0.004	0.009	0.017
Temperate steppe	0.373	0.000	0.015	0.008
Temperate desert steppe	0.202	0.009	0.042	0.008
*Spring*				
Temperate grasslands	0.071	0.031	0.090	0.036
Temperate meadow	0.177	0.008	0.075	0.032
Temperate steppe	−0.098	0.029	0.090	0.035
Temperate desert steppe	0.101	0.051	0.119^*^	0.042
*Summer*				
Temperate grasslands	0.939	0.001	−0.008	0.018
Temperate meadow	1.396^*^	0.001	−0.016	0.036
Temperate steppe	0.967	−0.001	−0.008	0.016
Temperate desert steppe	0.581	0.006	0.003	0.013
*Autumn*				
Temperate grasslands	0.389	0.004	−0.014	−0.009
Temperate meadow	0.529	−0.015	−0.013	0.002
Temperate steppe	0.399	−0.014	−0.020	−0.006
Temperate desert steppe	0.292	0.003	0.018	0.004
*Winter*				
Temperate grasslands	0.001	−0.008	−0.002	−0.013
Temperate meadow	−0.054	0.009	0.002	0.009
Temperate steppe	0.004	−0.005	−0.002	−0.008
Temperate desert steppe	0.018	−0.079	−0.005	−0.037

Significant at ^*^p < 0.05 and ^**^p < 0.01 levels.

## Discussion

### Spatiotemporal Changes in NPP of China’s Temperate Grasslands

Results showed that the NPP of China’s temperate grasslands significantly increased by 4.0 gC/m^2^/year (Figure 3) over the past 21 years. This result confirms that the vegetation productivity of temperate grasslands in China is increasing (Shen et al., 2015). The NPP of temperate meadow showed the largest increase. This result is similar to Guo et al. (2021) who found that the annual NPP showed the largest increasing trend for temperate meadow in Inner Mongolia from 2001 to 2018. However, there was a slight difference between the two studies. Guo et al. (2021) found that the increase of NPP for temperate meadow in Inner Mongolia was approximately 2.3 gC/m^2^/year, but the increase of NPP for temperate meadow in this study reached 5.4 gC/m^2^/year. In recent decades, human activities have caused grassland degradation and a decreasing trend in NPP of temperate grasslands (Jiang et al., 2021). Unlike the study of Guo et al. (2021), this study only analyzed unchanged grassland extracted from two land use/land cover datasets, which excluded, to some extent, the impacts of human activities on grassland vegetation. Therefore, this may account for the larger increasing trend of grassland NPP in this study than that obtained by Guo et al. (2021). In addition, based on field measured biomass data, Guo et al. (2021) found the average annual NPP of temperate meadow was about 391.1 gC/m^2^, which was larger than the average annual NPP of temperate meadow (316.9 gC/m^2^) in this study. Therefore, the different measurement methods may also partly explain the differences in trends of NPP between this study and Guo et al. (2021). However, future researches are still needed to further investigate how much of the difference of NPP is caused by measurement methods, and quantify the inaccuracies that caused by the measurement of satellite remote sensing.

### Climate Change Effects on NPP of China’s Temperate Grasslands

Over the entire temperate grasslands in China, we discovered that the annual precipitation had a significant positive correlation with the annual NPP (Table 1), which suggests that the increased precipitation could significantly increase the annual NPP of temperate grasslands. The significant positive correlation between precipitation and grassland NPP confirms our first hypothesis that the increase of precipitation is linked to the increase of NPP in the temperate grassland region of China. This is in agreement with Niu et al. (2008), who found that precipitation was a key factor limiting grassland vegetation growth in Inner Mongolia. The main reason may be that water conditions seriously limit grassland vegetation growth in warm and arid regions (Zhang et al., 2011; Jia et al., 2015). Increasing precipitation can promote vegetation growth by increasing soil water availability in arid regions, thus increasing the annual NPP of temperate grasslands (Guo et al., 2021). In addition, the NPP of different grassland types was significantly positively related to summer precipitation (Table 2), indicating that increasing summer precipitation could increase grassland NPP. Because summer is the season with the highest temperature in the study area, the continuous accumulation of high temperatures could intensify the evaporation of water in the soil and reduce water use efficiency in arid areas (Du et al., 2021; Guo et al., 2021; Shen et al., 2021); therefore, grassland vegetation has the most significant demand for water in summer (Guo et al., 2021). With an increase in summer precipitation, the photosynthesis of vegetation and soil water conditions can be improved (Piao et al., 2006), which leads to an increase in the annual NPP. In terms of the temperature effects, spring temperature has a positive relationship with grassland NPP of different types, and the increase in spring minimum temperature could increase the NPP of temperate desert steppe (Table 1). This is mainly because the temperate desert steppe region in the northwest of the study area has a much colder climate (Shen et al., 2018), and increasing the minimum temperature could decrease frost risk and thus promote the grassland vegetation growth (Gao et al., 2009).

Furthermore, we observed that summer minimum and maximum temperatures had asymmetric correlations with the NPP of temperate meadow. Specifically, nighttime warming could increase the NPP, whereas daytime warming could reduce the NPP of temperate meadow (Figures 6C,D). Our results confirmed the conclusions of Peng et al. (2013), who found an asymmetric effect of growing season temperature on the Northern Hemisphere’s grassland. However, our results revealed that the asymmetric relationship of temperature with NPP in China’s temperate grasslands was only found for temperate meadow in summer. This may be because, although nighttime warming can enhance autotrophic respiration and organic matter consumption, it can produce more organic matter by compensating and promoting photosynthesis the next day, thus promoting the growth of temperate meadow vegetation (Wan et al., 2009; Peng et al., 2013; Yang et al., 2016). According to relevant studies, rich nutrition and water are ideal environmental conditions for overcompensation (Belsky, 1986). Compared with other grassland types, temperate meadow is located in the northeast of the study area and has a humid to semi-humid monsoon climate with relatively more abundant water content (Piao et al., 2006; Shen et al., 2018). Simultaneously, better hydrothermal conditions in summer promote the occurrence of a compensation effect (Shen et al., 2018; Sun et al., 2022). Therefore, an increase in the minimum summer temperature is conducive to an increasing trend in NPP of temperate meadow. In addition, the NPP of temperate meadow had a significantly positive relationship with precipitation in summer, confirming that moisture is the key limiting factor for vegetation growth in the arid temperate grassland region. Therefore, increased summer maximum temperature can adversely affect grasslands by increasing evaporation (Liu et al., 2019). The negative correlation between grassland NPP and summer maximum temperature confirms our second hypothesis that the increase of daytime temperature may reduce the grassland NPP in warm season. Consequently, increased maximum temperature could inhibit the growth of grassland vegetation and reduce the NPP of temperate meadow.

During the study period of 2000–2020, the annual precipitation showed a significant increase trend in the study area (Table 3). According to the relationships between the NPP and meteorological factors (Table 2), we can conclude that the significant increase of precipitation could partly account for the increased NPP of temperate grasslands. Regarding the spatial changes of NPP, the largest increasing trend of NPP was found in the northeast study area. It is interesting that we also found the largest precipitation increase in the temperate meadow region over the past 21 years (Figures 2B, 7A). According to the spatial correlation results, increasing precipitation is likely to explain the increase in NPP of temperate meadow (Table 2 and Figures 4A, 7A). By contrast, the slight decreasing trend was concentrated in the western part of the study area (Figure 2B). Due to the negative relationships between the NPP and the temperatures (mean and minimum), the increases of mean and minimum temperatures may be the reasons for the decreased NPP of temperate desert grassland. Additionally, we observed that precipitation increased and temperature decreased in the southwest study area (Figure 7), which could partly account for the increased NPP of the temperate steppe from 2000 to 2020.

### Uncertainty

Although this study provided a comprehensive analysis of NPP changes and climate effects in temperate grasslands of China, there may still be some limitations. First, owing to the uncertainty of satellite remote sensing data, NPP data could contain some inaccuracies which may affect the results of this study. Second, the interpolation method of meteorological data in this study may lead to some inaccuracy in meteorological results, which could have an impact on the analysis of the results of this study. In addition, many factors influence vegetation growth in China’s temperate grassland. This study only considered two meteorological factors, temperature and precipitation, and the relationships of grassland NPP with other environmental factors need to be further explored. Finally, changes in NPP are affected by both climate change and human activities. Although this study extracted unchanged grassland, the influences of human activities on grassland vegetation could not be completely excluded. In addition, the results of this study are not verified by field measured data. Therefore, in future studies, the effects of human activities on temperate grasslands and the inaccuracies caused by the measurement of satellite remote sensing data should be further quantified.

## Conclusion

The annual NPP over the entire temperate grasslands in China increased by 4.0 gC/m^2^/year from 2000 to 2020, and the most significant increases were mainly concentrated in the eastern of China’s temperate grassland. For different grassland types, the annual NPP significantly increased by 4.3, 2.3, and 5.4 gC/m^2^/year for temperate steppe, desert steppe and meadow, respectively. In terms of climate impacts, we found increased annual precipitation can significantly increase the grassland NPP for different vegetation types. In contrast, increased annual temperature could result in a decrease of grassland NPP. For the effects of seasonal precipitation, precipitation in summer and autumn has a positive relationship with grassland NPP of three vegetation types. For seasonal temperature effects, increasing summer maximum temperatures could decrease the NPP of temperate grasslands. However, the increase of spring maximum was linked to the increase in NPP of temperate desert steppe. In addition, we first found asymmetric relationships of the NPP of temperate meadow with nighttime and daytime temperature in summer. Increased maximum temperature in summer reduced the NPP of temperate meadow, but warming minimum temperature in summer could increase the NPP of temperate meadow. In the context of global climate change, the influences of seasonal climate changes on grassland NPP for different vegetation types should be fully considered inaccurately simulating and predicting the vegetation productivity of temperate grasslands in China.

## Data Availability Statement

The original contributions presented in the study are included in the article/**Supplementary Material**, further inquiries can be directed to the corresponding author.

## Author Contributions

XS coordinated the project. RM carried out the data analysis and wrote the manuscript. CX, YL, YW, JZ, XL, and MJ contributed to modify the manuscript. All authors contributed to the article and approved the submitted version.

## Funding

This study was funded by National Natural Science Foundation of China (41971065), Youth Innovation Promotion Association, CAS (2019235), Key Research Program of Frontier Sciences, CAS (ZDBS-LY-7019), Natural Science Foundation of Jilin Province (20210101104JC), and Innovation Team Project of Northeast Institute of Geography and Agroecology, Chinese Academy of Sciences (2022CXTD02).

## Conflict of Interest

The authors declare that the research was conducted in the absence of any commercial or financial relationships that could be construed as a potential conflict of interest.

## Publisher’s Note

All claims expressed in this article are solely those of the authors and do not necessarily represent those of their affiliated organizations, or those of the publisher, the editors and the reviewers. Any product that may be evaluated in this article, or claim that may be made by its manufacturer, is not guaranteed or endorsed by the publisher.

## Footnotes

^1^ https://earthdata.nasa.gov/

^2^ http://data.cma.cn/

^3^ https://www.resdc.cn

## Supplementary Material

Supplementary Table 1 Information about climate data in 76 weather stations.

Supplementary Figure 1 Correlations between annual net primary productivity (NPP) and autumn precipitation **(A)**, autumn mean temperature **(B)**, autumn maximum temperature **(C)**, and autumn minimum temperature **(D)** of temperate grasslands in China during 2000–2020.

Supplementary Figure 2 Correlations between annual net primary productivity (NPP) and winter precipitation **(A)**, winter mean temperature **(B)**, winter maximum temperature **(C)**, and winter minimum temperature **(D)** of temperate grasslands in China during 2000–2020.
