# Supplementary figures and images for: Spatiotemporal Change of Net Primary Productivity and Its Response to Climate Change in Temperate Grasslands of China
Source: Front Plant Sci. 2022 May 24;13:899800. doi: 10.3389/fpls.2022.899800 (PMC9171389; doi:10.3389/fpls.2022.899800)

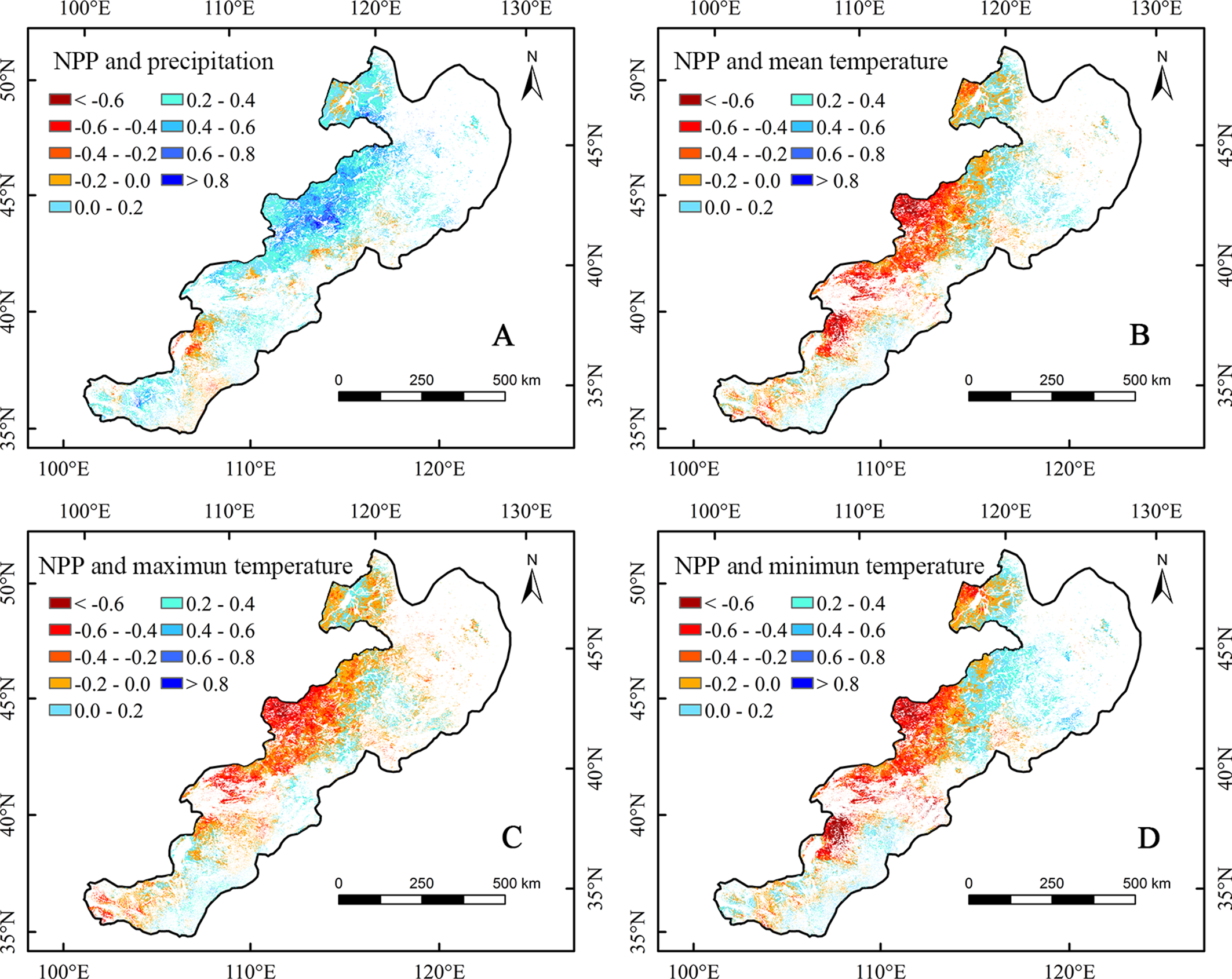

Supplement: Supplementary Figure 1 — Correlations between annual net primary productivity (NPP) and autumn precipitation (A), autumn mean temperature (B), autumn maximum temperature (C), and autumn minimum temperature (D) of temperate grasslands in China during 2000–2020. [file Image_1.TIF]

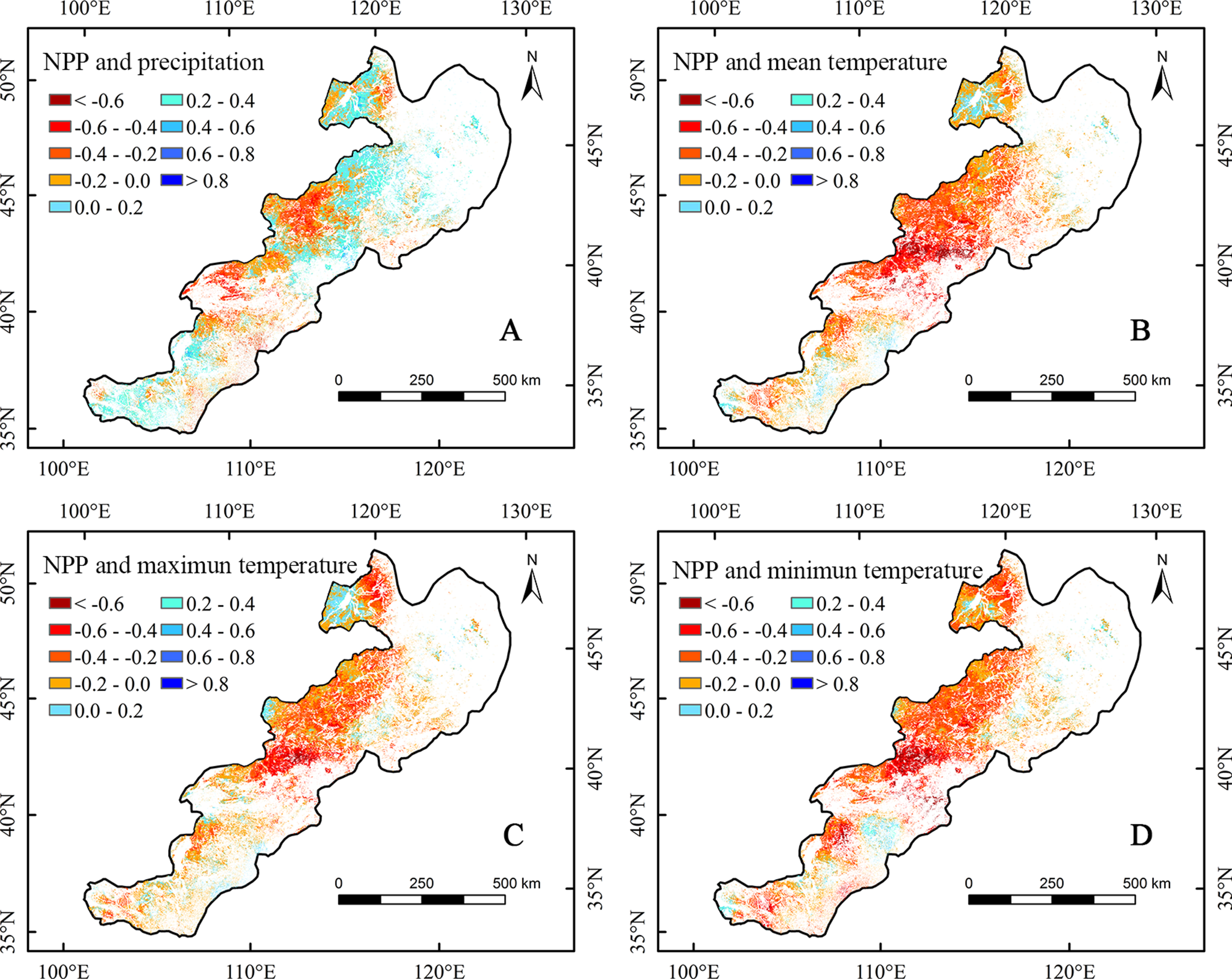

Supplement: Supplementary Figure 2 — Correlations between annual net primary productivity (NPP) and winter precipitation (A), winter mean temperature (B), winter maximum temperature (C), and winter minimum temperature (D) of temperate grasslands in China during 2000–2020. [file Image_2.TIF]
